# Clinical Syndromic Phenotypes and the Potential Role of Genetics in Pulmonary Vein Stenosis

**DOI:** 10.3390/children8020128

**Published:** 2021-02-10

**Authors:** Abbas H. Zaidi, Jessica M. Yamada, David T. Miller, Kerry McEnaney, Christina Ireland, Amy E. Roberts, Kimberlee Gauvreau, Kathy J. Jenkins, Ming Hui Chen

**Affiliations:** 1 Department of Cardiology, Boston Children’s Hospital, Boston, MA 02115, USA; AbbasHaider.Zaidi@cardio.chboston.org (A.H.Z.); Kerry.McEnaney@childrens.harvard.edu (K.M.); Christina.Ireland@cardio.chboston.org (C.I.); Amy.Roberts@cardio.chboston.org (A.E.R.); Kimberlee.Gauvreau@cardio.chboston.org (K.G.); kathy.jenkins@cardio.chboston.org (K.J.J.); 2 Department of Pediatrics, Harvard Medical School, Boston, MA 02115, USA; David.Miller2@childrens.harvard.edu; 3 Department of Pediatrics, Division of Genetics and Genomics, Boston Children’s Hospital, Boston, MA 02115, USA; Jessica.Yamada@childrens.harvard.edu

**Keywords:** pulmonary vein stenosis, genetics, trisomy 21, common atrioventricular canal, congenital heart disease, pulmonary hypertension, Down’s syndrome

## Abstract

Pulmonary vein stenosis (PVS) is a rare, frequently lethal disease with heterogeneous phenotypes and an unclear etiology. Limited studies have reported associations between PVS and congenital heart disease (CHD), chronic lung disease (CLD), and/or prematurity; however, to date, there have been no studies that report detailed clinical syndromic phenotypes and the potential role of genetics in PVS. An existing registry of multivessel PVS patients seen at Boston Children’s Hospital (BCH) was queried between August 2006 and January 2017 for all existing genetic testing data on these patients. PVS was defined as an intraluminal pulmonary venous obstruction in ≥2 vessels with mean pressure gradients > 4 mmHg. One-hundred-and-fifty-seven patients (46% female, with a median age at PVS diagnosis of 3 months) formed the cohort. Seventy-one (45%) patients had available genetic testing information. Of the 71 patients, a likely genetic diagnosis was found in 23 (32%) patients: 13 (57%) were diagnosed with Trisomy 21 (T21), five (22%) with Smith–Lemli–Opitz Syndrome, five (22%) had other pathologic genetic disease, and 24 (33%) had variants of unknown significance. The majority of 13 patients with T21 and PVS had common atrioventricular canal (CAVC) (10, 77%) and all had severe pulmonary hypertension (PHTN), which led to their PVS diagnosis. In our study, PVS was associated with T21, the majority of whom also had CAVC and PHTN. Therefore, complete assessment of the pulmonary veins should be considered for all T21 patients, especially those with CAVC presenting with PHTN. Furthermore, prospective standardized genetic testing with detailed clinical phenotyping may prove informative about potential genetic etiologies of PVS.

## 1. Introduction

Pulmonary vein stenosis (PVS) is a very rare and complex disease with a reported prevalence of ~1.7/100,000 in children younger than 2 years of age [1]. PVS in infants and young children can lead to pulmonary hypertension and right heart failure. If left untreated, PVS rapidly progresses and is fatal within months of diagnosis [2]. Unfortunately, despite advances in our understanding of the pathology and management of this disease, PVS presents on a wide spectrum, with different patients displaying a diverse array of symptoms, features, and co-morbidities, making detailed clinical characterization difficult. Furthermore, little is known about the etiology of this complicated disease. Multiple etiological factors and associations have been proposed to contribute to the development of PVS. These include mechanical abnormalities, from pathologic flow dynamics and/or specific anatomical features that trigger the development of PVS especially in children with congenital heart disease [3,4]. Furthermore, environmental factors such as prematurity and associated chronic lung disease may also play a role in the development of PVS [4,5,6]. Despite these factors being commonly present in patients, PVS remains rare, suggesting that there also may be a genetic basis of disease.

With the increasing sophistication and availability of genetic testing, many forms of congenital heart disease (CHD) have been shown to have a genetic component. However, to our knowledge, only a few studies have explored the possibility of a genetic component in PVS development, with detailed accompanying clinical characterization of patients. A previous retrospective case series from our institution demonstrated an association between PVS and Smith–Lemli–Opitz syndrome (SLO; OMIM# 270400), raising the possibility of a link between genetic disease and PVS [7]. Small case reports have also suggested an association of PVS with Trisomy 21 (T21) in a few patients [4,8]. However, a systematic review of the detailed clinical phenotyping of PVS patients with T21, their frequency, and the clinical factors associated with PVS has not been performed.

Overall genetic data in patients with PVS are scant, partly due to the rarity of the disease; moreover, patients with PVS do not routinely undergo genetic testing. Therefore, a more thorough clinical phenotyping of patients with multivessel PVS and examination of the available genetic information may begin to shed some light on the etiology of this complex disease. Therefore, in this study, we seek to characterize the clinical phenotypes, associated congenital heart disease, and genetics of patients from the PVS registry at Boston Children’s Hospital (BCH), one of the largest referral centers for this disease.

## 2. Materials and Methods

### 2.1. PVS Registry

We conducted a retrospective review of clinical and genetic data extracted from our institutional PVS registry. This registry was developed as part of a clinical trial to investigate the application of a multimodal treatment including antiproliferative tyrosine kinase blockade therapy in patients with significant multivessel disease [9]. PVS in our study was defined as an intraluminal pulmonary venous obstruction in ≥2 vessels with mean pressure gradients > 4 mmHg based on cardiac catheterization and echocardiography. We queried our institutional PVS registry for patients who met the above criteria between August 2006 and January 2017, and all clinical and genetic data were reviewed and extracted. The study was conducted in accordance with International Conference on Harmonization guidelines on Good Clinical Practice (ICH-GCP). Ethical approval was granted by the Boston Children’s Hospital Institutional Review Board (ID: M05-05-117; initial approval: 9 May 2005; updated approval: May 2020).

### 2.2. Statistical Analysis

Patient characteristics were compared for individuals (a) with and without available genetic information, (b) with and without congenital heart disease, and (c) with and without Trisomy 21. Continuous variables are summarized with medians and ranges and compared between groups with use of the Wilcoxon rank sum test. Categorical variables are displayed as frequencies and percentages and compared with Fisher’s exact test. Survival from date of PVS diagnosis until death or last follow-up was compared between groups using the log-rank test. Analyses were performed in Stata version 16 (StataCorp LLC); a *p* value <0.05 was considered statistically significant.

## 3. Results

### 3.1. Subject Demographics

**Figure 1:**
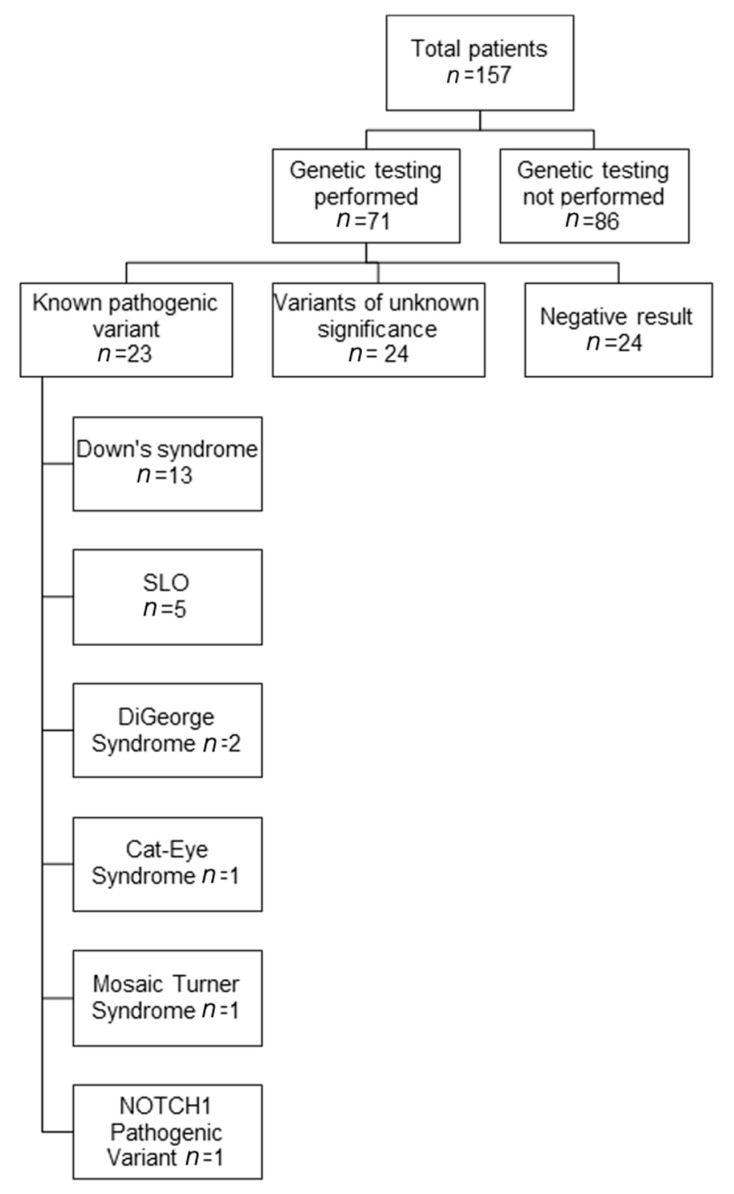
Available genetic testing results in patients with pulmonary vein stenosis (PVS). The genetic results of patients with known pathogenic variants are shown. Abbreviations: SLO, Smith–Lemli–Opitz syndrome.

A total of 157 patients (43% female) with median age at PVS diagnosis of 3 months (range: 1 day to 38 months) formed our cohort (Figure 1). Median follow-up time was 23 months (range: 1 day to 144 months). At the end of follow-up, 63 (40%) patients had died (Table 1).

### 3.2. Genetic Testing Results

Seventy-one (45%) of the 157 patients with PVS had genetic testing information available. There was no significant difference in PVS-associated clinical characteristics between patients who had undergone genetic testing, and those who did not (Table 1). Typically, genetic testing was performed when a physician had a clinical suspicion of an underlying genetic cause, but not routinely in all patients. Some patients also presented at our institution with an established genetic diagnosis. Genetic testing included karyotyping (*n* = 5), chromosomal microarray analysis (CMA, *n* = 26), fluorescence in situ hybridization for 22q11.2 deletion (FISH, *n* = 1), deletion/duplication analysis for single genes (*n* = 7), targeted gene sequencing (*n* = 7), disease-associated panels (*n* = 2), and clinical exome sequencing (*n* = 4). Pathogenic variants were found in 23 (32%) of 71 patients tested (Figure 1). Of the remaining patients, 24 (34%) were found to have variants of unknown significance (VUS), and 24 (34%) were found to have no abnormalities based on limited testing. A full detail of all identified genetic variants, along with clinical data, is presented in Table 2 and Table 3, and Supplemental Table S1. In PVS patients with pathogenic variants or VUS, multi-organ system involvement was usually seen. More than half of these children above had developmental delay and gastrointestinal abnormalities. Other organs that were commonly involved included the lungs, the brain, and the nervous and endocrine systems. Overall, in this large cohort of PVS patients, a large proportion (*n* = 23) were found to have syndromes which are generally known to have a genetic basis. Thirteen patients had T21, and five patients had SLOS which had been previously reported by Prosnitz [7], and another five had other pathogenic variants including 22q11 deletions or duplications.

Of the 71 patients who had genetic testing, many had multi-system involvement: 26 (37%) exhibited developmental delay, 19 (27%) had facial dysmorphism, 19 (27%) had gastrointestinal issues, 17 (24%) had musculoskeletal abnormalities, 15 (21%) had pulmonary disease, 14 (20%) had neurological issues, 14 (20%) had endocrine issues, 10 (14%) had genitourinary anomalies, seven (10%) had hematological issues, and five (7%) had renal disease. Please refer to Table 2 and Table 3 and Supplemental Table S1 for full details.

### 3.3. Congenital Heart Disease

In addition to PVS, 133 of the 157 (85%) patients had some form of congenital heart disease (CHD). The types of CHD included anomalous pulmonary venous connections (APVC) in 50 (32%) patients, complete atrioventricular canal (CAVC) in 21 (13%) patients, and hypoplastic left heart syndrome (HLHS) in 18 (11%) patients (Table 4). We also reported isolated atrial septal defect (ASD), ventricular septal defect (VSD), and patent ductus arteriosus (PDA) without other forms of CHD, and did not tabulate these lesions when they were associated with other forms of CHD. Importantly, 24 (15%) patients had isolated PVS with no other structural CHD. Patients with isolated PVS were more likely to be premature and have chronic lung disease (CLD), as compared to those with concomitant CHD (Table 5).

### 3.4. Trisomy 21 (n = 13)

Patient characteristics with T21 and detailed clinical phenotyping are presented in Table 2 and Table 6. The majority of patients had CAVC (10, 77%), which was notably more common than in PVS patients without T21 (*p* < 0.001) (Table 6). There was no difference in traditional PVS risk factors such as age at PVS diagnosis, gender, prematurity, chronic lung disease, or survival between patients with or without T21 (Table 6). PVS in children with T21 predominantly involved the upper pulmonary veins and left-sided pulmonary veins. Importantly, all T21 patients had severe pulmonary hypertension (Table 2). In approximately one-third of these T21 patients (*n* = 4), the development and diagnosis of PVS predated their CAVC repair. Five patients were diagnosed with PVS at least one month after their CAVC repair. In all patients, PVS was diagnosed due to persistence of severe pulmonary hypertension (PHTN) on clinical follow-up. In addition to structural cardiac disease in PVS patients with T21, many patients also had involvement of other organ systems. Ten patients (77%) also had pulmonary issues, including chronic lung disease (*n* = 8) and obstructive sleep apnea (*n* = 2), 11 (85%) patients had gastrointestinal involvement or feeding issues, necessitating G-tube placement (*n* = 5), gastroesophageal reflux disease (*n* = 3), and necrotizing enterocolitis (*n* = 2). Finally, three of these 13 patients had renal abnormalities, and three had hematological issues (Table 2).

## 4. Discussion

This study reports the genetic testing results from one of the largest cohorts of children with multivessel PVS. Furthermore, we provide detailed genotype–phenotype correlation for PVS patients who had underwent genetic testing. Nearly one-third of the cohort (*n* = 47/157) either had a genetic syndrome, a pathogenic variant, or a VUS that are associated with multi-organ abnormalities; these findings suggest an underlying genetic contribution for PVS.

We found that our multivessel PVS cohort was enriched for several genetically-linked syndromes. The most common genetic syndrome found in children with PVS was T21 which was seen in 13 patients. The second most common genetic syndrome was SLO, which was seen in five patients as previously reported by our institution [7]. Other syndromes seen in our patients include DiGeorge syndrome (OMIM# 188400), Cat-eye syndrome (OMIM# 115470), mosaic Turner syndrome, and Adams–Oliver syndrome-5 (OMIM# 616028) confirmed by a pathogenic variant in *NOTCH1*.

Importantly, our findings strengthen and further extend prior case reports where PVS has been associated with T21 (Supplemental Table S2). With our cohort, the total number of patients that are reported to have both PVS and T21 nearly doubles. We identified that CAVC was the most common type of structural heart disease found in children with PVS and T21. In our cohort, T21 patients with PVS all had significant pulmonary hypertension, which frequently led to their PVS diagnosis. Interestingly, our clinical phenotyping revealed that patients with T21 and PVS also had multi-system pathogenic involvement, again suggesting a genetic contribution to PVS (Table 2). Furthermore, recent work demonstrated that abnormal flow patterns in the pulmonary veins due to increased left-to-right shunt flow also contribute to development of PVS [10]. Therefore, it may be that the combination of genetics with mechanical, environmental, and clinical factors together lead to the vascular phenotype of PVS.

In addition to the known pathogenic variants found in this cohort, we provide the most extensive collection of genetic information and clinical phenotyping for PVS patients with variants of unknown significance (VUS). This may serve as a resource for future studies and work in the area.

### 4.1. Takeaways

Our findings suggest that T21 patients with CAVC and severe PHTN should be assessed for PVS. This diagnosis, though rare, may contribute to PHTN that is refractory to standard treatment. Additionally, aside from T21 patients, the clinical phenotypes and genetic information presented for known pathogenic genetic variants may prove useful for clinicians considering PVS as a potential diagnosis in patients with certain forms of CHD and clinical features (Table 3). We recommend that clinicians consider genetic testing for PVS patients, especially those with multi-system abnormalities. Clinicians considering a genetic consult or genetic testing for their PVS patients may wish to use the genetic data in Table 2 and Table 3, and Supplemental Table S1 as a reference or starting point.

### 4.2. Limitations

Our study was limited by the amount of genetic information and clinical data available due to its retrospective nature. This study also was limited by the inherent selection biases of a patient referral population. Our PVS registry included patients with multivessel PVS and therefore represents those who are more severely affected and the study results may not be generalizable for those with single vessel or milder PVS disease.

Another limitation is that about half of the patients in our PVS registry did not undergo any genetic phenotyping. Despite this, a large number (23/157, 15%) of the entire cohort had a clinical diagnosis of a genetic syndrome, with most of those confirmed by genetic testing to identify the pathogenic variant. Notably, however, there were no significant differences in patients with or without available genetic testing in terms of patient demographics such as age, gender, prematurity, the prevalence of CLD, or survival (Table 1). Therefore, if routine comprehensive genetic testing were implemented for patients with multivessel PVS, one could hypothesize that genetic abnormalities may likely be present in at least the same or larger percentage of PVS patients than in our cohort. The heterogeneity of genetic variants identified in this cohort suggests that clinical exome sequencing, including copy number variant detection, should be considered for individuals presenting with a diagnosis of PVS.

## 5. Conclusions

In summary, the most common genetic association of multivessel PVS in our registry was Down’s syndrome (T21) with CAVC, followed by Smith–Lemli–Opitz syndrome. We also found multi-organ abnormalities in children with PVS who were determined to have genetic variants of unknown significance. Our study suggests that genetics should be considered as a potential factor in the development of PVS in children. Prospective systematic genetic testing may be useful in elucidating the complex etiologies of this rare disease.

## Figures and Tables

**Table 1:** General characteristics of patients with PVS and comparison of patients with and without available genetic information (*n* = 157).

	Total (*n* = 157)	Available Genetic Information (*n* = 71)	No Available Genetic Information (*n* = 86)	*p*-Value
Age, mo, median (range)	3 (1 day to 38 mo)	3 (1 day to 22 mo)	3 (1 day to 38 mo)	0.93
Female sex, *n* (%)	67 (43%)	33 (46%)	34 (40%)	0.42
Prematurity, *n* (%)	53 (34%)	26 (37%)	27 (31%)	0.50
Congenital heart disease, *n* (%)	133 (85%)	57 (80%)	76 (88%)	0.19
Chronic lung disease	54 (34%)	25 (35%)	29 (34%)	0.87
Died, *n* (%)	63 (40%)	32 (45%)	31 (36%)	0.32
Follow-up, mo, median (range)	23 (1 day to 144 mo)	23 (1 day to 144 mo)	23 (4 days to 143 mo)	0.78

Data are presented as range, number (percentage). Abbreviations: mo, months.

**Table 2:** Clinical phenotyping of PVS patients with Trisomy 21 (*n* = 13).

Sex	PVS Location	Dx Age (mo) Pre/Post Surgery	Pre-MatureY/*N*	CHD	Cardiac Surgery	Pulmonary	GI	Neuro	Endocrine	Renal	Heme	Other
M	RUPV, LUPV stenosis	16 Pre	N	CAVC	PA banding, BiV and PVS repair	Sev. PHTN, OSA	Poor weight gain, s/p G-tube	Dev. delay				
M	RMPV & LLPV stenosis RUPV & LUPV atresia	4 Post	Y (35w)	CAVC	CAVC repair, sutureless PV repair	Sev. PHTN, CLD, laryngomalacia, vocal cord surg.	NEC s/p small bowel resection for strictures, GERD, G-tube	Dev. delay	Hypothyroid			Cleft palate
F	Mild RUPV, LUPV, & LLPV stenosis	2	Y (29w)	VSD	None	Sev. PHTN, CLD	NEC s/p bowel resection, short gut syndrome, liver failure	Dev. delay, brain hemorrhage	Hypothyroid	Renal failure	Leukopenia, thrombocyto- penia, anemia	Auto- amputation of digits
M	LUPV, LLPV, & RMPV stenosis, RUPV atresia	5	Y (29w)	Large PDA	Bilateral sutureless PVS repair	Sev. PHTN, CLD	Imperforate anus s/p ileostomy	Dev. delay	Hypothyroid			
F	LUPV stenosis, RUPV atresia	0 Pre	Y (30w)	CAVC	CAVC repair	Sev. PHTN, CLD	G-tube	Dev. delay			Capillary leak	
M	LUPV & LLPV stenosis	4 Post	N	Large PDA	PDA ligation	Sev. PHTN	Poor feeding, aspiration	Dev. delay		Hydrone-phrosis		
F	LLPV stenosis, LUPV atresia	0 Pre	N	CAVC	CAVC repair, sutureless PV repair	Sev. PHTN		Dev. delay				
F	All 4 PV	3 Post	Y (28w)	CAVC	CAVC repair, PVS repair	Sev. PHTN, CLD, CDH s/p repair		Dev. delay	Hypothyroid			Chronic otitis media
M	All 4 PV	6 Post	N	CAVC	CAVC and VSD repair, sutureless PVS repair	Sev. PHTN, CLD, OSA		Dev. delay		VUR, Recurrent UTIs		
M	RUPV atresia, LUPV & LLPV stenosis	5 Post	N	CAVC	CAVC repair, PVS repair	Sev. PHTN	GERD	Dev. delay				
F	RUPV, LUPV, & RMPV stenosis	3 Pre	N	CAVC	CAVC and PVS repair	Sev. PHTN, CLD	GERD, chronic aspiration	Dev. delay				
F	All 4 PV	2 Post	N	CAVC	CAVC repair, PVS repair	Sev. PHTN	G-tube	Dev. delay				
M	RUPV, LUPV, & LLPV stenosis	4 Post	N	CAVC	CAVC repair, sutureless PVS repair	Sev. PHTN, CLD	G-tube recurrent GI bleed	Dev. delay			Thrombocytopenia	

Abbreviations: BiV, biventricular; CAVC, common atrioventricular canal; CHD, congenital heart disease; CDH, congenital diaphragmatic hernia; CLD, chronic lung disease; Dev. Delay, developmental delay; F, female; GERD, gastroesophageal reflux disease; GI, gastrointestinal; Heme, hematologic; LLPV, left lower pulmonary vein; LUPV, left upper pulmonary vein; M, male; N, no; NEC, necrotizing enterocolitis; Neuro, neurologic; OSA, obstructive sleep apnea; PA, pulmonary artery; PDA, patent ductus arteriosus; PHTN, pulmonary hypertension; PV, pulmonary vein; PVS, pulmonary vein stenosis; RMPV, right middle pulmonary vein; RUPV, right upper pulmonary vein; s/p, status post; Sev., severe; Surg., surgery; UTI, urinary tract infection; VSD, ventricular septal defect; VUR, vesicoureteral reflux; W, weeks; Y, yes. Note. Blank boxes in the table indicate no reported abnormalities.

**Table 3:** Clinical phenotyping of PVS patients with other pathogenic variants (*n* = 10).

Sex	Genetic Testing Results	Dysmorphic Features	Musculo- skeletal	Pulm	GI	Neurologic/ Developmental Delay	Endocrine	Renal	GU	Heme	Other
F	SLOS: *DHCR7* c.964-1 G > C *DHCR7* c.1138T > C (p.Cys380Arg)	X	X			X		X	X		Webbed toes
M	SLOS: Elevated 7-dehydrocholesterol (=157 μg/mL)			X	X	X	X				Vision impair
M	SLOS: *DHCR7* c.964-1G > C *DHCR7* c.1210C > A (p.Arg404Ser)		X		X	X	X		X		
M	SLOS: *DHCR7* c.964-1 G > C *DHCR7* c.976G > T (p.Val326Leu)		X		X	X			X		Bilateral hand polydactyly, webbed toes
M	SLOS: *DHCR7* c.964-1 G > C *DHCR7* c.1138T > C (p.Cys380Arg)										
F	Mosaic Turner Syndrome			X	X	X		X		X	Retinopathy of prematurity
F	22q11 deletion—coord. uk	X		X	X	X	X				DiGeorge syndrome
F	22q11 deletion (min: 18914689-21461788, max: 18913560-21797456, hg19)	X			X	X	X			X	Anemia
M	22q11 duplication—coord. uk				X						Cat eye syndrome
F	*NOTCH1* p.2448dupC (p.C817LfsX11)		X			X	X	X	X		

Abbreviations: Bil, bilateral; coord. uk; coordinates unknown, GI, gastrointestinal; GU, genitourinary; Heme, hematological; Pulm, pulmonary; SLOS, Smith–Lemli–Opitz Syndrome. Note, Blank boxes in the table indicate no reported abnormalities, X in boxes indicate reported abnormalities.

**Table 4:** Structural heart disease in PVS patients.

Type of Heart Disease	*n* = 157 (%) ^1^
Anomalous PV connection	50 (32%)
CAVC	21 (13%)
Isolated ASD	21 (13%)
HLHS	18 (11%)
Heterotaxy syndrome	15 (10%)
Isolated VSD	13 (8%)
Dextrocardia	9 (6%)
Tricuspid atresia	7 (4%)
Isolated PDA	7 (4%)
Coarctation of aorta	6 (4%)
DORV	6 (4%)
PA	6 (4%)
Scimitar syndrome	4 (3%)
Valvar PS	4 (3%)
TGA	4 (3%)
Single RV	3 (2%)
Single LV	3 (2%)
Cor Triatriatum	3 (2%)
TOF	1 (1%)

^1^ As patients may have more than one type of CHD, percentages sum to greater than 100%. Abbreviations: ASD, atrial septal defect; CAVC, common atrioventricular canal; DORV, double outlet right ventricle; HLHS, hypoplastic left heart syndrome; LV, left ventricle; PA, pulmonary atresia; PDA, patent ductus arteriosus; PS, pulmonary stenosis; PV, pulmonary venous; RV, right ventricle; TGA, transposition of the great arteries; TOF, Tetralogy of Fallot; VSD, ventricular septal defect.

**Table 5:** Comparison of PVS patients with and without congenital heart disease.

	CHD (*n* = 133)	Non-CHD (*n* = 24)	*p*-Value
Age, mo, median (range)	4 (1 day–38 mo)	5 (1–33)	0.28
Female sex, *n* (%)	61 (46%)	6 (25%)	0.073
Premature, *n* (%)	39 (29%)	14 (58%)	0.009
Chronic lung disease, *n* (%)	38 (29%)	16 (67%)	<0.001
Died, *n* (%)	50 (37%)	13 (54%)	0.16
Follow up, mo, median (range)	23 (4 days–144 mo)	9 (1 day–83 mo)	0.035

Data are presented as range, number (percentage). Abbreviations: CHD, congenital heart disease; mo, months.

**Table 6:** Comparison of PVS patients with and without Trisomy 21.

	Trisomy 21 (*n* = 13)	Non-Trisomy 21 (*n* = 144)	*p*-Value
Age, mo, median (range)	4 (1 day–16 mo)	3 (1 day–38 mo)	0.87
Female sex, *n* (%)	6 (46%)	61 (42%)	0.78
Premature, *n* (%)	5 (38%)	48 (33%)	0.76
Congenital heart disease, *n* (%)	12 (92%)	121 (84%)	0.69
Chronic lung disease, *n* (%)	6 (46%)	48 (33%)	0.37
Died, *n* (%)	5 (38%)	58 (40%)	0.80
Follow up, mo, median (range)	21 (1-46)	23 (1 day–144 mo)	0.31
Cardiac diagnosis, *n* (%)			
CAVC	10 (77%)	11 (8%)	<0.001
Isolated lesions			
ASD	0 (0%)	21 (15%)	0.22
VSD	1 (8%)	12 (8%)	1.0
PDA	1 (8%)	6 (4%)	0.46
PVS	1 (8%)	23 (16%)	0.69
Pulmonary vein involvement, *n* (%)			
RU	11 (85%)	101 (70%)	0.35
RL	4 (31%)	81 (56%)	0.09
LU	12 (92%)	127 (88%)	1.0
LL	9 (69%)	122 (85%)	0.23

Abbreviations: ASD, atrial septal defect; CAVC, common atrioventricular canal; LL, left lower; LU, left upper; mo, month; PDA, patent ductus arteriosus; PVS, pulmonary vein stenosis; RL, right lower; RU, right upper; VSD, ventricular septal defect.

## Data Availability

The data presented in this study are available on request from the corresponding author. The data are not publicly available in order to maintain patient privacy.

## Supplementary Materials

The following are available online at https://www.mdpi.com/2227-9067/8/2/128/s1, Table S1: Clinical phenotyping of PVS patients with genetic variants of unknown significance (*n* = 24); Table S2: Published patients with Trisomy 21 and PVS (*n* = 17).
