# Supplementary material for: Clinical Syndromic Phenotypes and the Potential Role of Genetics in Pulmonary Vein Stenosis
Source: Children (Basel). 2021 Feb 10;8(2):128. doi: 10.3390/children8020128 (PMC7916466; doi:10.3390/children8020128)
Supplement: Supplementary file 1 [file children-08-00128-s001.pdf]

Supplementary

Table S1. Clinical phenotyping of PVS patients with genetic variants of unknown significance (n = 24).

Sex	Genetic Testing Results	Dysmorphic features	Musculo-skeletal	Pulm	GI	Neurologic/ Developmental delay	Endocrine	Renal	GU	Heme	Other
F	Xp11.4-q12 - absence of heterozygosity (26.5-26.6 Mb) min: 40505730-66956221,<br>max: 40505730-6714630 (build unknown)<br>RECQL4 c1573delT (p.Cys525Alafs*33)<br>RECQL4 c1568G>C (p.Ser523Thr)				X	X	X				
M	*MTHFR* variant – coord. uk			X	X	X				X	
M	*TJP2* c.1845dupT(p.3616*)<br>ABCB11/PFTC2 c.2093G>A (p.Arg698His)	X	X		X	X	X			X	
F	2p12-16.1 duplication - min: 58818430-78868846,<br>max: 58641848-78889090 (hg19)<br>Xp11.22-q13.1 (area of homozygosity/copy number neutral) - min: 50357704-68569057, max: 50357704-68754736 (hg19)	X	X			X	X		X		
F	16p11.2 duplication (3 copies) (718-818 kb) - min: 29478119-30196611, max: 29378488-30196631 (hg19)	X		X	X	X					Bil. lung tx
M	1p33 deletion (111 kb) - 48626183-48737445 (build unknown)	X	X			X			X		
F	*SETD5* c.3497G>A (p.Trp1166Ter)<br>*CYP21A2* c.955C>T (p.Gln319Ter)	X	X	X	X	X			X		Lung tx candidate

	FANCA c.1115-1118delTTGG (p.Val372Alafs*42)								
M	Xq27.2-q28 duplication – coord. uk	X	X	X	X	X	X	X	
F	2q21.1 duplication - 130700489-130867257 (hg18)<br>5q33.3 duplication - 157099532-157131554 (hg18)<br>8q22.3 duplication - 104543169-104655550 (hg18)<br>10p11.21 duplication - 35418295-35485580 (hg18)	X		X		X			
F	15q24.2-26.3 inverted duplication - coordinates unknown<br>47, XXY, + inverted duplication (15) (q24qter) [18]/46, XX [2] - coord. uk	X	X	X		X			
M	2p22.1 deletion (217 kb) - 40096481-40313076 (hg36?)	X				X		X	Optic nerve hypoplasia
F	14q12-q21.1 deletion - min: 31150741-38710155, max: 2948044-39694531)<br>46, XX, t(3;14)(q22;q13) - coord. uk	X	X	X	X	X	X		Bil. 5th finger clinodactyly
F	Ring chromosome 18<br>18p11.22-pter deletion - coord. uk<br>18q21.1-21.31 duplication - coord. uk<br>18q21.31-ter deletion - coord. uk	X	X			X	X		Electrolyte issues, ankyloglossia
F	46, XX, (t8;16)(p;p) - Trisomy of short arm of 16, monosomy of short arm of 8 - site uk			X	X	X			
M	*RBM8A* c.-12G>A<br>*TRIP11* c5719+2T>C								

F	2p13.2 deletion - 73706727-73764497 (hg18)<br>5q22.1 deletion - 109663569-109775919 (hg18)	X	X			X		X			Right eye coloboma
M	2q23.2-q23.3 duplication - 150381658-151146949 (hg18)<br>1q36.32 duplication - 2409400-2436580 (hg18)<br>3p26.1 duplication - 4814075-4850199 (hg18)<br>12q13.12 deletion - 47419972-47446900 (hg18)	X	X			X	X				Strabismus
M	3q28 duplication (4 copies) (468-528 kb) - min: 189963519-190431505, max: 189922604-190450439 (hg18)	X	X	X	X	X	X				
M	5p15.33 duplication - coord. uk<br>15q24.3-q26.1 deletion - 75484918-88086130 (coord. uk)									X	Anemia
F	15q11.2 deletion - coord. uk			X	X	X	X				
M	*CRELD1*: p.Arg329Cys	X	X	X	X	X		X	X	X	Left groin hematoma
M	*KCTD13* variant - site uk					X					
M	F5 c.1691G>A (p.Arg506Gln)	X	X	X	X	X				X	Asplenia
M	Heterozygous Cystic Fibrosis variant, clinically negative for Cystic Fibrosis										

Abbreviations: Bil, bilateral, coord., coordinates; GI, gastrointestinal; GU, genitourinary; Heme, hematological; Pulm, pulmonary; Tx, transplant; uk, unknown. Note. Blank boxes in the table indicate no reported abnormalities.

Table S2. Published patients with Trisomy 21 and PVS (n = 17).

Author	Year	Patient	Age at Dx (mo)	M/F	Associated Cardiac Dz	PV Involvement (No.)	Additional Notes
**Tandon and Edwards**	1973	1	unknown	unknown	AVSD	3	RUPV, LUPV, LLPV
**Stewart**	1992	2	9	M	Secundum ASD	2	RUPV, LUPV
		3	2.5	F	Large VSD, small PFO	3	
**Chakrabarti**	2007	4	7	M	PDA	4	
		5	7	F	None	2	RUPV, LUPV
**Vaideeswar**	2008	6	1	M	None	CPV	
		7	1 day	F	VSD, aortic atresia	CPV	
**Drossner**	2008	8	12	unknown	ASD, VSD, PDA	2	
		9	6.5	unknown	AVSD, PDA	1	
		10	4	unknown	AVSD	1	
		11	35	unknown	ASD	2	
		12	2	unknown	None	3	
**Gowda**	2013	13	7	F	Small ASD, PHTN	2	RUPV, LUPV
		14	6	M	PDA, ASD, prosthetic MV	2	LUPV, LLPV, HF, PHTN
		15	5	M	CAVC	3	RUPV, LUPV, LLPV, hypothyroid, respiratory distress
**Mahadevaiah**	2015	16	3	M	HCM, CAVC, PDA, PFO	2	RUPV, LUPV, chronic lung dz
**Zahari**	2017	17	3		Dilated RA/RV, ASD, RCA fistula, PHTN	4	Respiratory distress, myeloproliferative dz

Abbreviations: AVSD, atrioventricular septal defects; ASD, atrial septal defect; CPV, common pulmonary vein; Dx, diagnosis; Dz, disease; HCM, hypertrophic cardiomyopathy; HF, heart failure; LLPV, left lower pulmonary vein; LUPV, left upper pulmonary vein; Mo, months; MV, mitral valve; PDA, patent ductus arteriosus; PFO, patient foramen ovale; PHTN, pulmonary hypertension; RA, right atrium; RCA, right coronary artery; RUPV, right upper pulmonary vein; RV, right ventricle; VSD, ventricular septal defect.

References for Supplementary Table 2

Tandon, R.; Edwards, J.E. Cardiac malformations associated with Down's syndrome. Circulation 1973, 47(6), 1349-1355.

Stewart, A.D.; Calder, A.L.; Neutze, J.M.; James, A.H.; Brandt, P.W. Stenosis of pulmonary veins in Down syndrome. J Paediatr Child Health 1992, 28(2), 192-195.

Chakrabarti, S.; Mittal, R.; Gnanapragasam, J.P.; Martin, R.P. Acquired stenosis of normally connected pulmonary veins. Cardiol Young 2007, 17(3), 322-327.

Vaideeswar, P.; Tullu, M.S.; Sathe, P.A.; Nanavati, R. Atresia of the common pulmonary vein--a rare congenital anomaly. Congenit Heart Dis 2008, 3(6), 431-434.

Drossner, D.M.; Kim, D.W.; Maher, K.O.; Mahle, W.T. Pulmonary vein stenosis: prematurity and associated conditions. Pediatrics 2008, 122(3), e656-661.

Gowda, S.; Bhat, D.; Feng, Z.; Chang, C.H.; Ross, R.D. Pulmonary vein stenosis with Down syndrome: a rare and frequently fatal cause of pulmonary hypertension in infants and children. Congenit Heart Dis 2014, 9(3), E90-97.

Mahadevaiah G, Gupta M, Ashwath R. Down Syndrome with Complete Atrioventricular Septal Defect, Hypertrophic Cardiomyopathy, and Pulmonary Vein Stenosis. Tex Heart Inst J. 2015;42:458–461.

Zahari N, Sirisani JDD. Progressive pulmonary vein stenosis in Down syndrome infant: a rare cause of pulmonary hypertension. Med J Malaysia. 2018;73:2.
